# St. Louis Medical Society

**Published:** 1858-06

**Authors:** 


### *St Louis Medical Society.*

Dr. W. M. McPheeters reported to the Society a case of Unusual Rigidity of the Perineum.—This case, said he, was interesting in many respects, especially in reference to the obstetrical procedure which was resorted to, for its relief.

At 9 o'clock, A. M., he was called to a lady then in labor with the first child. Things had proceeded on slowly but regularly, and in the evening, the child's head had fairly descended into the pelvic cavity; at midnight, the head reached the perineum, which it considerably distended; the pains then were so frequent and so powerful, the distension of the perineum was such that at every moment he expected to see the issue of the child's head. However, notwithstanding the energetic action of the uterus continued without intermission, no progress was perceptible. Fearing at this juncture an imminent and extensive rupture of the perineum, he had recourse to venesection to the amount of thirty ounces, to the warm bath, to fomentations, ect.; the state of nausea in which the patient was for several hours, contra-indicated the use of antimonials. In spite of all these means, the rigidity of the perineum gave no signs of yielding, and in order to deliver the patient, he feared that no alternative was then left him except to lessen the child's head. However, before resorting to this extreme measure, he thought that he would try another means. It was to incise the perineum. This he did by introducing a guarded bistoury at the margin of the extended vulva and performing an incision in the perineum in a diagonal direction, sufficient in length to procure the desired opening; the opening was hardly completed, when, with strong expulsive pains, the child's head made its appearance externally, and was soon followed by the shoulders and the rest of the body. He thought this result very encouraging, and although not aware that this operation had been performed before, he would certainly have recourse to it again under the same circumstances, and would recommend it to others.

He would further remark, that the child was born alive and that the patient recovered without any untoward symptom.

Dr. Pallen said, that in his opinion, Dr. McPheeters, under the above circumstances, had adopted a most judicious course of conduct. Not only was the operation warrantable, but it had been recommended by the highest authorities, such as Dr. Blundell. He had himself taught this procedure and applied it in similar cases for many years. Lacerations of the perineum were, said he, very much to be deplored and should be prevented with the utmost care. This laceration may extend sometimes very far, involving the sphincter ani, etc. However, in a case of that description, he had observed an admirable proviso of nature, which consisted in the establishment of another internal sphincter for the anus above the seat of laceration, thus preventing the disastrous consequences of this lesion under ordinary circumstances.

Dr. Hammer, in reference to the case related by Dr. McPheeters, said, that the procedure he adopted in this instance was a regular obstetrical operation taught and practised in Germany for many years. Naegele, whom he heard lecturing on this very subject, speaks of this operation as having been of long use in Germany. Dr. Hammer added, that in order to make the incision fulfil its object, which was the enlargement of the vulva, the incision should be sometimes rather extensive and deep. If the incision, said he, was not sufficient, the pressure of the child's head would certainly continue it and produce thus a ragged cut instead of the clean incission which the knife would perform. He would remark, however, that it is a thing of rare occurrence that the distension of the perineum be so great as to necessitate this operation. By referring to the statistics of the large hospitals, we seldom notice this operation mentioned. In Germany, forceps are generally used in such cases. Their action is to alter the form of the child's head, by elongating it and thus facilitating its passage. Moreover, lessening the rigidity of the perineal muscles by other means; among these, chloroform by inhalations or local fomentations. Kneading the perineum, he said, would also often be of benefit. Killian, of Bonn, recommends this method.

Dr. Pallen, in reply to the above remark, said, that he thought that the applications of the forceps would have here increased instead of diminishing the difficulties. The forceps, said he, are compressors of the head only to a very limited degree, as shown by Baudelocque. The forcep is mainly an instrument for extraction, a *tire-tete*. Chloroform, said he, was advisable for many reasons. It does relieve spasmodic muscular action; he had tried it many times with marked success in cases where the version was indicated, and where it would have been next to impossible without the relaxing influence of this powerful anæsthetic. The kneading, spoken of, merits also, said he commendation; he had practised many years this method, which he once learned from Dr. Linlon.

Dr. McPheeters replied, that he was well convinced that in this case an application of the forceps would inevitably have caused a rupture of the perineum. He had often applied them under similar circumstances, but not when there was that degree of rigidity; he added, that fomentations and inunction of oil had been freely used; the patient was already naturally nauseated so as to preclude the use of antimony, she had been bled to a considerable amount, and the co-aptation of the head to the perineum was so intimate that the very act of introducing the forceps would have produced laceration. Furthermore, there were expulsive pains enough without the forceps. Chloroform was not administered on account of the repugnance of the patient's friends to it. Moreover, he considered that the very pressure of the child's head was the greatest relaxant that could have been devised; and all the above means, after a fair trial, proved of no avail. But after the incision made in the perineum, the child's head issued without the slightest difficulty.

Dr. Hammer considered the variety of opinion regarding the use of the forceps in these cases, as a mere matter of discrepancy between two continental schools. In England and America, craniotomy, said he, was outrageously frequent, whilst in France and Germany, forceps operations were preferred to the former more bloody procedure. The Germans consider that a certain degree of disproportion between the child's head and the pelvis or the perineum may be overcome by a judicious use of the forceps. However, the disproportion is sometimes so great that it can not be overcome. Under the circumstances of Dr. McPheeter's case, the Germans would have used the forceps; there was, it is said, an impossibility of introducing the instrument during the pains, but in the absence of a pain this might have been done. It has been said that the rigidity of the perineum is principally kept up by the presence of the child's head; but the spasmodic contractions of the perineum thus induced, alternate with those of the uterus. By the action of the forceps this alteration is annulled by the continued pressure it allows to be made on the perineum with the child's head, whilst it is embraced in the blades of the forceps.

Naegele, although of a timid temperament, in other things, used the forceps liberally.

The forceps, by compressing the child's head, elongated it. This instrument did not perhaps much diminish the size of the child's head, but by altering its shape it facilitated its passage through a narrow pelvis.—Did not nature proceed in the same manner in cases of disproportion between the head and the pelvis? In those cases it was to be remarked that the head was sometimes considerably elongated. In this wise did the action of the forceps imitate the operation of nature.

Dr. McPheeters admitted the possibility of this elongation. As for using the forceps in this case, he considered it was utterly impossible either during a pain or in its absence; for the fitting of the head to perineum was so close the patient complained loudly of the mere introduction of the finger, which she compared to a cutting instrument.

As to the spasmodic contractility of the perineum referred to by Dr. Hammer, it was altogether destroyed in this case, as will occur whenever the muscular fibre has been acting powerfully for a length of time.

Dr. M. M. Pallen thought that so far as this case was concerned, the delay of the passage of the child's head through the vulva was not owing in any degree to inertia of the uterus or to a want of the *vis a tergo*; this existed to a sufficient extent to have procured the birth of the child under ordinary circumstances; but here was a most rigid perinieum against which the child battered in vain—a danger of laceration was imminent as the result of natural forces alone—the forceps, he thought would have increased the danger of this laceration. A great deal of prudence should guide the accoucheur in deciding upon the cases where the forceps would be useful. He was himself a strong advocate of the forceps under certain circumstances, although he must say that he condemned the use of the *long* forceps under any circumstance. In this, he was aware that he differed from the opinions taught by all accoucheurs in America, and especially in Germany. He was not, however, inclined to undervalue the great and valuable progress made in the art of obstetrics by the Germans; he knew full well that Naegele had done more than any man, except Baudelocque, to advance this art. But whilst he acknowledged their eminence he did not in everything adopt the teaching of that school; he endorsed the doctrines taught in London and Dublin; he was convinced from statistics, that more women recovered after craniotomy, than after the use of forceps when applied above the pelvis. The mortality of the children was also very great in those cases; frequently, also, various ruptures, and also vesico or recto-vaginal fistulæ were the result of the imprudent applications of the forceps. Sieblod had, said he, applied the forceps once in every seven cases of midwifery which occurred in his practice. This preference of the Germans for the forceps, was, he said, not based upon merely scientific reasons, but resulted in a great degree from peculiar religious prejudices.

Dr. Hammer, in reply, denied that the German accoucheurs were influenced by peculiar religious opinions in this matter: their adoption of the forceps, in many cases where it was not employed by the English, was based upon purely scientific grounds. In Germany, the forceps was better studied and understood in all its applications, and selected, therefore, as a very safe and useful instrument in cases where the English or American accoucheur would, without hesitation, sacrifice the child's life. In Germany, in those cases, they tried to save the child and mother also, and they, generally succeed. In England, one of the reasons why the forceps were not used, was on account of that mock modesty so peculiarly British, which did not allow of a woman to be uncovered. Acting on this false modesty they have not encouraged the establishment of lying-in hospitals to the same extent that they have done on the continent. They do not understand the forceps for want of facilities to study its applications, hence their hesitation in applying it. The Germans, who are familiar with that instrument, are more bold in its employment.

As for the action of the forceps in the case of Dr. McPheeters, he thought that the rigidity of the perineum was owing to a spasmodic contraction of the muscles of that part, comparable to the spasmodic strictures which occur sometimes in the urethra. If a bougie is introduced when one of these spasmodic strictures exists, it will impinge against it, without penetrating through it; but if the bougie be left there for a few minutes, the part will gradually become accustomed to it, and it will at length penetrate through the stricture. The same process will occur in spasmodic contraction of the perineum. This said he, was the rationale of the action of the forceps in those cases. If the child's head be pressed for some time by the forceps on a rigid perineum, the spasmodic contraction of that part will gradually yield. As for the ruptures and vesico or recto-vaginal fistulæ, which according to Dr. Pallen, were often the result of the use of the forceps, he thought that they took place oftener when the forceps were not used, as in those cases which have been left to nature or where craniotomy was performed. Vesico-vaginal fistulæ happen mostly in the protracted cases which have been left to nature. Statistics would prove this.

In the United States there are, said he, as many craniotomy cases as there are forceps cases in the rest of the world; because the forceps are not used at all in some cases where it could have been beneficially applied. in the beginning of the labor, and craniotomy is resorted to as a last resort. He would not blame the profession here for this state of things; it originated from the want of lying-in hospitals, as he had already remarked.

Dr. Pallen said, that he would see with the greatest satisfaction the establishment of lying-in hospitals in this country, and he expressed the hope that the Biddle Lying-in Hospital, in process of construction in this city, would some day prove a source of practical instruction to students desirous of perfecting themselves in the science of obstetrics. As for himself, he was far from condemning the use of forceps at the inferior strait; he was in the frequent practice of applying it himself—and he had taught its utility for years;—he would add, that his opinion was that it was not applied in many cases in this country where its usefulness would be incontestible if better understood, and where craniotomy was, unfurtunately, performed, especially in the country, where the practitioner is often left to himself.

Dr. Smith was one day present, said he, at a lecture on obstetric operations, delivered by Dr. Murphy, of London; in that lecture, he made the startling remark, that statistics would bear him out in the assertion that death oftener followed operations of craniotomy at the superior strait than cæsarean sections.

Dr. Smith said, that as for the rigidity of the perineum giving way under the continued pressure of the child's head, as asserted by Dr. Hammer, he would rather expect to see the rigidity increased under this prolonged pressure; he would expect to see inflammation setting in under these circumstances, and a spasmodic contraction firmly established. As for the procedure employed by Dr. McPheeters, in the case he had reported, he thought it perfectly warrantable and worthy of imitation under similar circumstancas.—*St. Louis Medical and Surgical Journal.*

